# Technical and analytical approach to biventricular pressure-volume loops in swine including a completely endovascular, percutaneous closed-chest large animal model

**DOI:** 10.1016/j.jvssci.2024.100190

**Published:** 2024-01-17

**Authors:** David P. Stonko, Mathieu C. Rousseau, Colin Price, Amy Benike, Rebecca N. Treffalls, Nichole E. Brunton, Dorian Rosen, Jonathan J. Morrison

**Affiliations:** a Division of Vascular Surgery and Endovascular Therapy, Department of Surgery, The Johns Hopkins Hospital, Baltimore, MD; b Division of Vascular and Endovascular Surgery, Mayo Clinic, Rochester, MN; c Division of Thoracic Surgery, Department of Surgery, University of Montreal, Montreal, QC, Canada; d School of Medicine, University of the Incarnate Word, San Antonio, TX; e Gonda Vascular Center, Mayo Clinic, Rochester, MN; f Tetrachromatic Science Consulting, Medford, MA

**Keywords:** Biventricular pressure-volume loops, LV PV loops, RV PV loops, Endovascular, Closed-chest

## Abstract

Pressure-volume (PV) loop analysis is a sophisticated invasive approach to quantifying load-dependent and independent measures of cardiac function. Biventricular (BV) PV loops allow left and right ventricular function to be quantified simultaneously and independently, which is important for conditions and certain physiologic states, such as ventricular decoupling or acute physiologic changes. BV PV loops can be performed in an entirely endovascular, percutaneous, and closed-chest setting. This technique is helpful in a survival animal model, as a percutaneous monitoring system during endovascular device experiments, or in cases where chest wall compliance is being tested or may be a confounder. In this article, we describe the end-to-end implementation of a completely endovascular, totally percutaneous, and closed-chest large animal model to obtain contemporaneous BV PV loops in 40 to 70 kg swine. We describe the associated surgical and technical challenges and our solutions to obtaining endovascular BV PV loops, closed-chest cardiac output, and stroke volume (including validation of the correction factor necessary for thermodilution), as well as how to perform endovascular inferior vena cava occlusion in this swine model. We also include techniques for data acquisition and analysis that are required for this method.

## Clinical Relevance

This article describes the end-to-end implementation of a completely endovascular, percutaneous, and closed-chest large animal model for obtaining biventricular pressure-volume loops in swine. This will allow researchers to obtain contemporaneous, continuous left and right ventricular physiology through a minimally invasive procedure that animals can survive and does not alter chest wall compliance. This model may be used to better quantify the physiology during clinical scenarios of ventricular decoupling, during procedures such as transcatheter aortic valve repair or those that alter left ventricular (LV) function, or during the initiation of resuscitative endovascular balloon occlusion of the aorta or extracorporeal cardiopulmonary resuscitation when LV and right ventricular filling become misaligned.

## Graphical abstract

**Figure:**
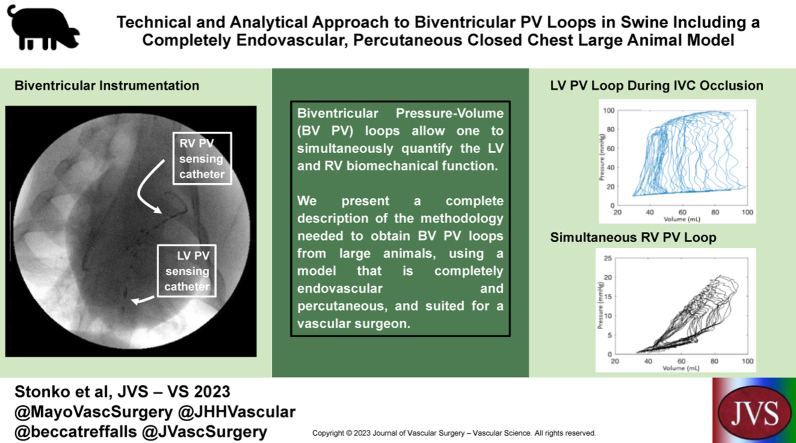

**Article Highlights**

• **Type of Research:** Large animal translational research study
• **Key Findings:** We present the end-to-end implementation of a completely endovascular and percutaneous large animal model to obtain contemporaneous biventricular pressure-volume data.
• **Take Home Message:** Endovascular surgeons may want to study the impact of their procedures or devices on cardiac function using large animals. This strategy provides sophisticated biventricular cardiac functional parameters while remaining entirely endovascular.

Pressure-volume (PV) loop analysis is a comprehensive approach to determining cardiac biomechanical function in translational research.^1^, ^2^, ^3^, ^4^ With a left ventricular (LV) PV loop, one can determine the load-dependent LV functional parameters, such as stroke volume (SV) or ejection fraction. Although these metrics can also be obtained with noninvasive strategies such as echocardiography, other imaging, or indirect measurements, PV loop analysis allows even further quantification of cardiac function. This includes the load-independent measures, namely the end-systolic and end-diastolic PV relationships, which are an “independent” measure of inotrope without the influence of cardiac preload.^5^, ^6^, ^7^, ^8^ The technique may hold promise for application in human subjects, such as monitoring during thoracic endovascular aortic repair and transcatheter aortic valve repair.

Careful strategies are required to obtain these high-fidelity PV loops. We have previously described a method to obtain and analyze LV PV loops, in addition to multibeat estimation of load-independent parameters without augmenting preload in a large animal model.^9^ In some scientific scenarios or clinical questions, performing a clamshell thoracotomy or sternotomy to obtain open-chest PV loops via apical puncture may be appropriate. However, in other scenarios, this may interfere with scientific data collection or confound the study results (eg, in survival swine surgery, for testing percutaneous medical devices, or when chest wall compliance must be maintained).

The potential opportunity set with PV loop analysis in large animals has recently prompted the industry to develop the next generation of PV loop acquisition systems.^10^, ^11^, ^12^, ^13^, ^14^ Central to this evolution has been the ability to obtain high-fidelity in vivo simultaneous biventricular (BV; contemporaneous LV and right ventricular [RV] loops) PV loops, which are now technically possible but not well described or validated in the literature for large animals. With previous technology, true BV PV loops were difficult to obtain in practice due to the added instrumentation (eg, catheter placement in the LV and RV) and the interference between LV and RV PV-sensing catheters because the systems operate at the same frequency.

Although BV PV loops are now technically attainable, significant challenges remain for vascular and endovascular translational research labs. There is a limited selection of endovascular catheters and wires that work with the PV-sensing systems and are appropriate for animal use. The catheters are not designed to be totally endovascular nor intended to be delivered or steered percutaneously. Specifically, ADInstruments has a 5F catheter and a 7F catheter that are supported by a wire but cannot be placed over a wire. Therefore, they both must be delivered directly. This can present issues when accessing the vessels, crossing the valves, and landing appropriately in the ventricle, as they are somewhat brittle, stiff, and not steerable. Furthermore, for the surgical implementation to be fully endovascular, the inferior vena cava (IVC) occlusion method and PV loop systems calibration with a known SV must also be fully endovascular. In an open-chest model, a flow probe on the aorta or main pulmonary artery (PA) can provide a cardiac output (CO) and SV. In a completely closed-chest model, we recommend thermodilution as a reliable, endovascular way to get accurate swine SV/CO, which is required by the BV PV loop model. Thermodilution is computed with a known “correction factor,” which is dependent on animal anatomy and experimental approach. The correction factor must be validated before implementing this endovascular large animal BV PV loop model.

This article aims to describe the end-to-end implementation of a completely endovascular, percutaneous, and closed-chest large animal model for obtaining BV PV loops for research. We describe the surgical and technical challenges and our solutions to obtain endovascular BV PV loops, closed-chest CO, and SV, including validation of the correction factor necessary for thermodilution, as well as how to perform IVC occlusion in swine ranging from 40 to 70 kg.

## Materials and Methods

The study was performed at the Mayo Clinic after the institutional animal care and use committee approval (IACUC protocol no. A00006990-23). The study was approved for the use of castrated male Yorkshire swine weighing between 40 and 70 kg, which is the primary species and size used in our laboratory for testing adult human-translatable physiology and human-designed surgical, medical, and endovascular devices. We have used these techniques in swine weighing up to 90 kg.^9^^,^^15^, ^16^, ^17^, ^18^, ^19^, ^20^, ^21^, ^22^, ^23^, ^24^, ^25^, ^26^, ^27^, ^28^ If swine weighing less than 40 kg are used, we recommend imaging their external iliac artery diameter to confirm that it will support instrumentation.^29^^,^^30^ We chose the gender of the animals based on availability and cost, and ultimately, only male animals were included in the study.

### Animal husbandry and preparation

All animals used in this study underwent our typical animal husbandry and preparation protocol.^19^^,^^20^ Briefly, all animals were initially sedated with telazol (4-5 mg/kg) and xylazine (1.8-2.2 mg/kg) via intramuscular injection. After sedation, they were given an intramuscular injection of buprenorphine hydrochloride (0.03 mg/kg). Animals were endotracheally intubated and maintained under inhalational anesthesia with isoflurane (1%-3%). The minimum alveolar concentration was titrated between 1.2 and 1.8. Mechanical ventilator settings were placed on volume-controlled mode with a tidal volume of 8 to 12 mL/kg, fraction of inspired oxygen 40% to 100%, and a respiratory rate titrated to maintain an end-tidal CO_2_ between 35 and 45 mm Hg.

To vary CO across a wide physiologic range for thermodilution validation, animals were treated sequentially with 50 mcg norepinephrine, 1 mg/kg esmolol, or 5 mcg/kg/min dobutamine. Timing of the onset of the drug effect was determined empirically, and return to baseline vitals was observed for at least 10 minutes before beginning another drug treatment.

Percutaneous vascular access was obtained for all described instrumentation, including at least the left carotid artery (7F to support the temperature-sensing catheter for the LV PV catheter), the right external jugular (7F for injection into the right cavoatrial junction during thermodilution, and then to support the RV PV-sensing catheter), and a femoral vein (7-14F to support the balloon-bearing catheter used for IVC occlusion) using a modified Seldinger technique. A solid-state pressure catheter (Transonic Corporation) was placed in an external iliac artery to measure continuous blood pressure via a 5F to 7F sheath. For the CO validation, 16- to 20-mm vascular probes (Transonic Corporation) were used. Two ADV 550 PV systems were used for the BV PV loop system, with 5F and 7F PV-sensing pigtail probes.^14^

**Fig 1:**
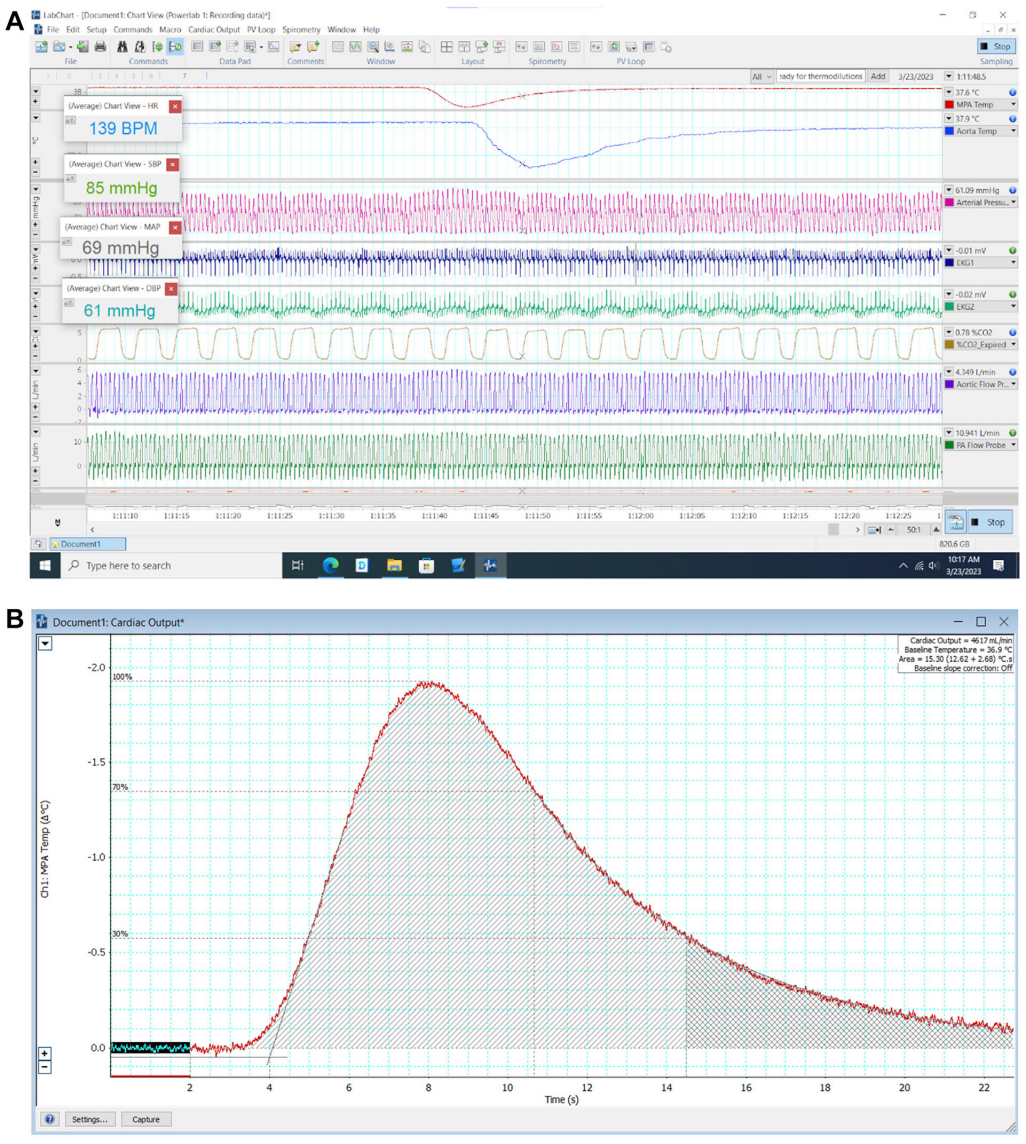
An example of an intraprocedural LabChart screen capture during a thermodilution. **A,** An intraprocedural LabChart view showing each of the data parameters being monitored and captured on the animal. Each row corresponds to one data channel capturing one parameter, which include blood temperature in the main pulmonary artery (*MPA*) (°C), blood temperature in the aortic arch (°C), arterial blood pressure (mm Hg), two electrocardiography (*EKG*) tracings, end-tidal CO_2_ (%), aortic flow (L/min), and MPA flow (L/min). The first two channels demonstrate an example of a thermodilution temperature augmentation as sensed by a thermistor in the MPA and aorta, respectively. **B,** The same temperature tracing shown in channel 1 in panel A, inverted and zoomed in as shown in the cardiac output module. *BPM*, Beats per minute.

Continuous physiological data acquisition was performed using the PowerLab system and observed and recorded in LabChart (ADInstruments). This included arterial blood pressure (mm Hg), heart rate (beats per minute), rectal temperature (°C), electrocardiography tracings (mV), and the aforementioned main PA and aortic blood temperatures and flows. An example of this is shown in real time, as observed bedside in the laboratory, in Fig 1, *A*.

### Statistical analysis

Statistical analysis was performed using Stata v17.0 (Stat Corp LLC) and GraphPad Prism v8.0 (GraphPad Software Inc). Statistical significance was determined a priori as *P* < .05. Data were presented as mean and standard deviation. Linear regression with R^2^ and 95% confidence bands were computed to evaluate the association of correction factor and CO. GraphPad Prism was used for the visual representation of data.

### Technical approach to endovascular biventricular PV loops

BV PV loops are obtained by placing a PV-sensing catheter into the LV and RV and obtaining data from them contemporaneously. Our laboratory uses a pair (one for the LV and one for the RV) of ADV550 systems, which are calibrated to two different frequencies so they do not interfere with one another. These can be placed directly by inserting them through the apex of the heart via myocardial puncture or by endovascularly introducing them to the ventricles.^11^^,^^31^^,^^32^ Open-chest strategies may be preferred in settings where endovascular procedures are not possible, for nonsurvival surgery where incision size may not be important, and in cases where the chest is already open for other reasons. A closed-chest endovascular strategy has obvious advantages with regard to cost and risk. It can also address scientific questions where maintaining chest wall compliance is important.

### Technical approach to totally closed-chest, BV PV loops

We first place the LV PV-sensing catheter and then place the RV PV-sensing catheter, both under image guidance. After the PV catheters are placed, we optimize their positions using fluoroscopy and confirm that the PV phase, magnitude, volume, and pressure are transmitted appropriately to LabChart.

### Instrumenting the LV

Swine anatomy underpins our percutaneous, endovascular closed-chest strategy to obtain PV loops. The swine aortic arch has a bicarotid trunk, which gives rise to the right subclavian artery and both carotid arteries.^30^ The carotids then travel cranially before dividing into the internal and external branches. The internal carotid continues into the rete mirabile before entering the circle of Willis. The rete mirabile can capture large emboli before they reach the cerebral circulation and, therefore, significantly reduces stroke risk as compared with humans and also introduces resistance to the internal carotid vascular circuit, which makes swine comparably more reliant on the vertebral for cerebral perfusion at baseline. If this technique were applied to humans, another access point may be more ideal to minimize the risk of stroke, such as femoral, brachial, or radial arteries.

This strategy builds on our previously published technical approaches to isolated LV PV loops.^9^ First, we obtain at least a 7F access via the Seldinger technique. Next, the 7F wire-supported PV-sensing catheter is inserted up to the valve with the wire fully deployed. We safely cross the valve with the wire pulled back 3 to 4 cm before carefully hubbing the catheter in the apex. This method may be altered to suit researchers’ needs, such as using 5F catheters in smaller animals.

### Instrumenting the RV

Obtaining RV access with the PV system is more difficult, and again, the anatomy underpins the technical strategy. Unlike humans, the swine RV does not fully extend down to the apex and is mainly constrained to the cranial aspect of the anterior heart. Supplementary Video 1, online only, contains a typical digital-subtracted angiogram that demonstrates this anatomy, with a catheter introduced from the right jugular, through the right atrium (RA), RV, and into the proximal main PA after an LV PV loop catheter has already been positioned in the apex from the carotid. Here, the catheter takes the anticipated trajectory of an anterior-posterior view from the right jugular, through the superior vena cava, into the RA, then through the center of the RV, and into the main PA. The RV and LV catheters cross one another in the middle of the heart. Although swine cardiac anatomy differs from human cardiac anatomy, the differences are minor, and accessing the RV in humans would take the same trajectory (eg, superior vena cava, RA, RV, and main PA).

**Fig 2:**
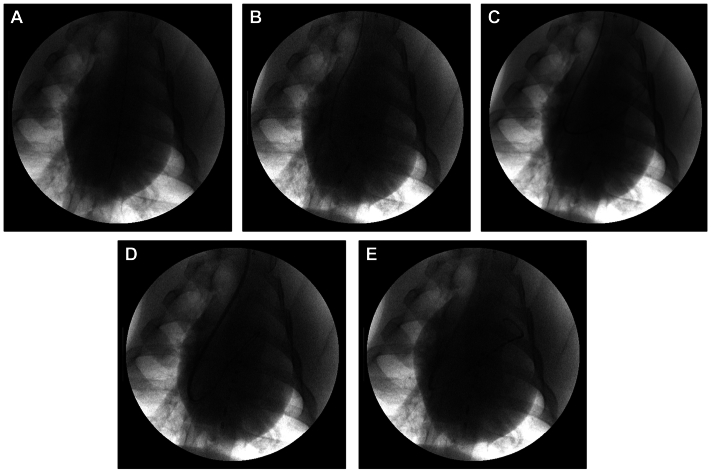
Fluoroscopic technical progression for reproducible placement of a right ventricular (RV) pressure-volume (PV)-sensing catheter. **A**, Anteroposterior view of the chest with the left ventricular (LV) PV loop already placed. **B**, Catheter access into the right atrium (RA). **C**, Wire and catheter access through the RV into the main pulmonary artery (PA). **D**. Upsized catheter with access maintained to the RV. **E**, Deployed RV PV-sensing catheter. Here, the support catheter maintains durable access while keeping the PV-sensing portion of the PV catheter straight through the center of the ventricle, which allows for reliable and appropriate PV acquisition.

This endovascular course can be quite difficult for rigid catheters to take. It is possible to directly introduce a 5F pigtail PV-sensing catheter from the right jugular and can drop this in and across the RV, but the catheters are not designed for such maneuvering. Furthermore, the catheter must take a j-curve, making it difficult to get a straight segment entirely within the RV and obtain a good signal. The limited selection of available catheters increases the difficulty and risk of injury or catheter breakage. We have developed a reproducible way of introducing these catheters and obtaining reliable RV PV loops that mitigate these concerns. Fig 2 demonstrates the introduction of an RV PV loop. Starting with an LV PV-sensing catheter from the right carotid, a 6F right external jugular access is obtained (Fig 2, *A*). A catheter (usually a 5F Davis, hockey-stick, or multipurpose catheter with a moderate angle) is introduced into the RV (Fig 2, *B*), and a glide catheter is advanced into the RV and the main PA (Fig 2, *C*). Once the wire is introduced, one may then upsize the catheter for at least a 6F catheter that will be used to support the PV-sensing catheter. In this case, we use a 6F Super Arrow-Flex (Arrow; Teleflex). This catheter was used to obtain Supplementary Video 1, online only, described above. This is our default catheter choice, but others could be used depending on the experimental design.

The next important step involves extending the 5F PV-sensing catheter through the supporting Super Arrow-Flex until it is fully in the RV. The support catheter is then retracted until the PV-sensing portion of the catheter is fully exposed, but not beyond this point. This allows the support catheter to keep the PV-sensing catheter straight within the center of the ventricle, preventing damage to the PV-sensing catheter. It is sometimes possible to remove the support catheter, resulting in a more gradual curve in the RV PV loop, such as in Supplementary Video 2, online only. In some animals, particularly smaller ones, an appropriate straight segment can be challenging to obtain without a support catheter. Fig 2, *E* shows a fully deployed 5F PV-sensing catheter in the RV, with the support catheter retracted to just beyond the j-curve, leaving a long straight segment of the PV-sensing component of the catheter in the RV. This is the ideal fluoroscopic positioning of the endovascular BV PV-sensing catheters.

Here, the LV PV loop is in the apex with a slight rightward curve, and the RV catheter crosses the LV catheter at the center of the heart, extended toward the pulmonary valve but not across it. Once this configuration is obtained, we confirm that pressure signals are clinically appropriate waveforms (eg, the RV catheter should transduce RV pressures, not PA pressures/waveform). Appropriate waveforms indicate that the system is working and that the catheters were not broken during the introduction. Finally, both catheters are positioned by optimizing the phase and magnitude signals. Towel clips are used to prevent the catheters from moving. This is especially important for the LV PV-sensing catheter, which can be slowly ejected over time by the LV contractions and is generally a good practice.

It is acceptable to place catheters using an alternative method by first steering catheters based on pressure tracings followed by optimization using phase/magnitude/volume tracings and confirming the position on plain film; in the same way, one may place a swan catheter using pressure transduction.^14^ Because of the stiffness of the catheters and difficulty crossing valves endovascularly without injury or catheter abuse, we favor image-guided placement in most percutaneous scenarios, followed by minor positional changes by signal optimization.

### IVC occlusion

**Fig 3:**
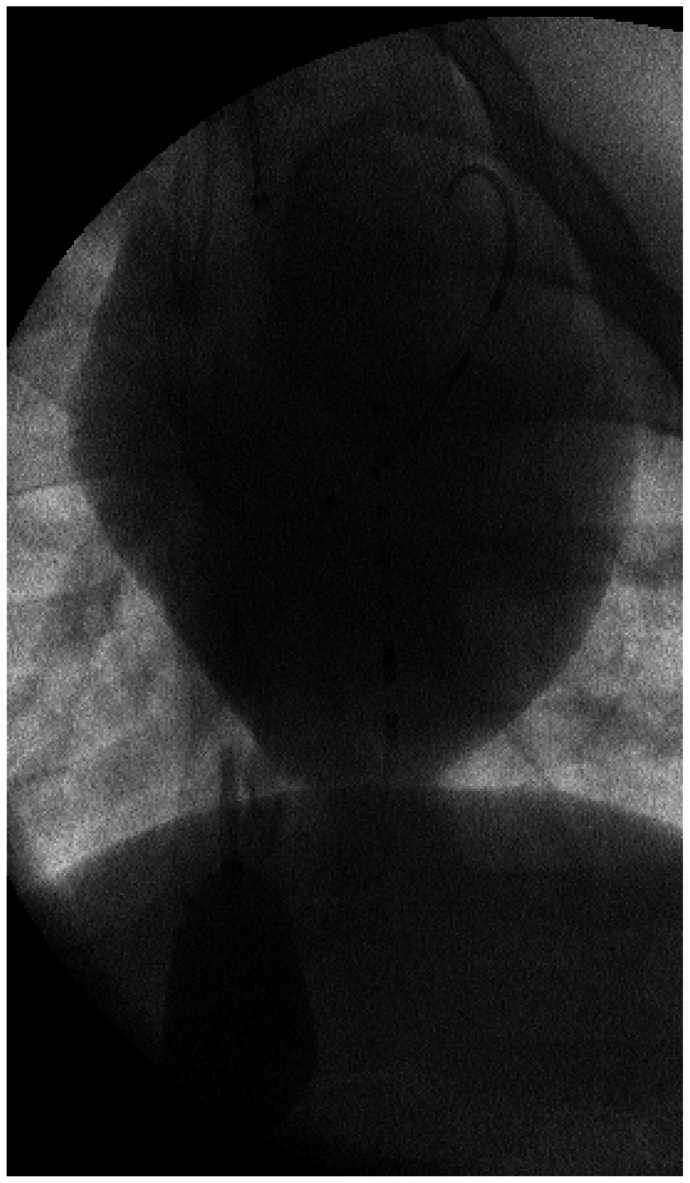
Fluoroscopic view of biventricular (BV) pressure-volume (PV) sensing catheters during an inferior vena cava (IVC) occlusion with a Coda balloon. A 7F wire-supported catheter is in the left ventricular (LV), with the tip positioned in the apex. A 5F pigtail right ventricular (RV) PV-sensing catheter is correctly placed in the middle of the RV, crossing the LV catheter in the middle of the heart and just below the level of the diaphragm, positioned to reduce preload from the IVC and the hepatic veins. Supplementary Video 2, online only, shows this same IVC occlusion in video format.

Regardless of how the PV-sensing catheters are placed, we use endovascular techniques to occlude the IVC and obtain load-independent metrics (Box 2). This is easily accomplished with femoral venous access and positioning of an occlusive balloon at or above the level of the hepatic vein confluence with the IVC. Fig 3 shows the typical positioning of a complete BV PV loop setup while undergoing an IVC occlusion with a Coda Balloon Catheter (Cook Medical). This is a semicompliant balloon, which can safely be inflated in the IVC and provide reliable occlusion. For survival surgery, we recommend using an ER-REBOA catheter (Prytime Medical Devices Inc) for IVC occlusion. This has the benefit of only requiring a 7F access and uses a compliant balloon, which is safe for IVC occlusion, and we have used it many times. A venogram can identify the inflow of the hepatic veins to the IVC (Fig 3, Supplementary Video 2, online only), but if this is not used or is impractical, the balloon can be positioned more cranially to confirm that the preload contributed by the IVC is abolished.

Box 2 **Procedural steps to perform endovascular inferior vena cava (IVC) occlusion to determine load-independent biventricular pressure-volume (PV) loop parameters.**

**Table:** 

1. Confirm that data acquisition is appropriate, and catheters are positioned correctly, and the technology is working. If necessary, correct laboratory or physiologic derangement before IVC occlusion.
2. Confirm that the balloon-bearing catheter is appropriately positioned in the IVC, at or cranial to the hepatic vein inflow.
3. In rapid sequence:
a. Start a breath hold, which should reduce artifact.
b. Go live or perform digital subtraction angiography on fluoroscopy.
c. Inflate the balloon at a rate of approximately 1 cc/s.
d. Monitor the PV loop migration on LabChart.
e. Once sufficiently migrated (generally approximately 5 seconds), deflate the balloon.
f. Resume respirations.

### Thermodilution and BV PV data acquisition and analysis

#### Thermodilution

PV-sensing catheters use a manometer to measure pressure directly. Volume is computed from the change in the electric field during the cardiac cycle. Specifically, the change in the electric field is the difference in phase and magnitude over time as the myocardium moves in and out of the electric field.^33^, ^34^, ^35^, ^36^, ^37^ This method requires a known SV for calibration. If there is not a concern regarding precise PV data or only relative changes in PV data, an estimate can be used instead for their species and weight choice. An accurate model requires precise SV, which can be obtained by using the described endovascular technique. In this model, the BV PV data would directly inherit any inaccuracy in SV estimation. Various methods, such as an echocardiogram and thermodilution, could be adapted to estimate SV if the technique is appropriately validated. We use thermodilution due to its high reliability. Thermodilution can also be performed with the same technologies already being used for the PV system (eg, LabChart with the CO module). The surgical instrumentation already being performed for the RV PV system makes this trivial from a technical standpoint and only takes a few minutes.

Performing thermodilution with the system requires creating a repeatable method for a given animal species and weight range. Our step-by-step approach to performing thermodilution is summarized in Box 1, and these have been described for many animal species previously. This method requires careful calibration and validation and is affected by animal species/strain, animal weight, and sensor locations. Once this is accomplished for a given animal model, it may be used for future close-chest studies.

Box 1 **Procedural steps to obtain cardiac output (CO) and stroke volume (SV) using thermodilution in 40 to 70 kg swine.**

**Table:** 

1. Place 30-cc saline syringes in a cold ice bath with a temperature probe to determine saline temperature.
2. Obtain vascular access (right internal jugular vein to inject at the right cavoatrial junction, and secondary access to deliver temperature probe, placed in the pulmonary artery).
3. Confirm placement fluoroscopically of all instruments and good data acquisition on LabChart.
4. Obtain saline temperature and then inject saline over 1 second.
5. Capture thermodilution temperature transit and run the cardiac output module on LabChart, using the established correction factor. An example of this temperature transit is shown in Fig 1, *A*, and CO computation in Fig 1, *B*.
6. Repeat this process to obtain triplicate values, confirming repeatedly stable CO estimation. Compute the median CO.
7. Using this CO and the heart rate, SV can be computed.

**Fig 4:**
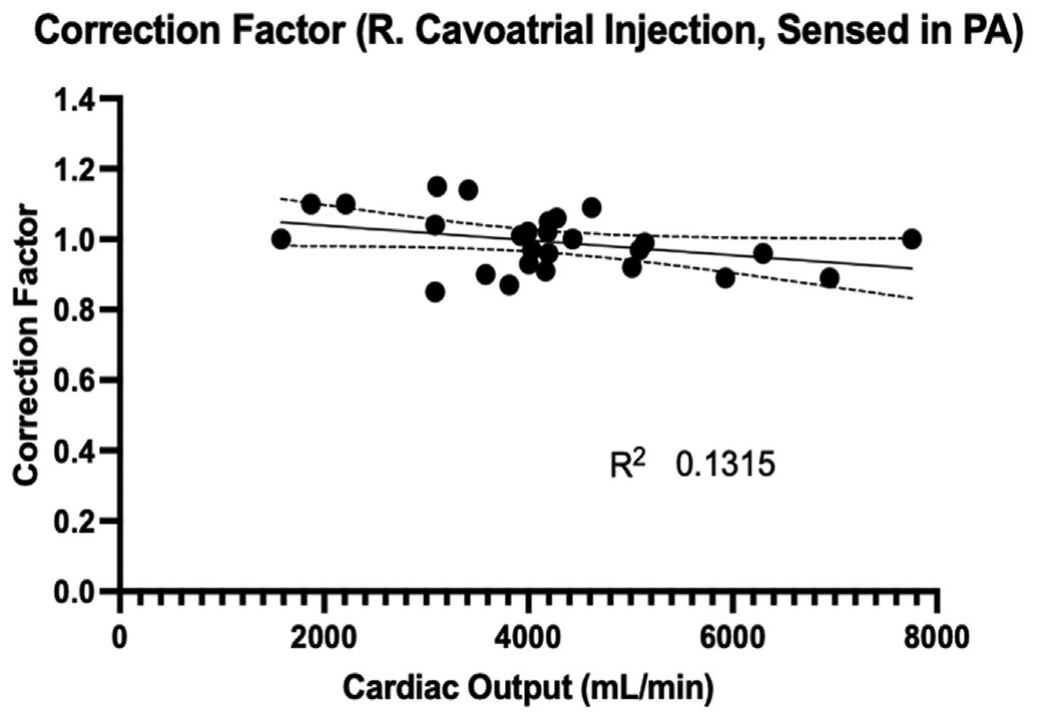
Determination of the correction factor as determined by thermodilution, compared against the cardiac output from a main pulmonary artery (*PA*) flow probe. The thermodilution involves the injection of 30 cc of cold saline into the right cavoatrial junction over 1 second, and sensed by a temperature probe in the PA.

Because our lab primarily uses 40 to 70 kg swine, we validated our method using three swine that weighed 45, 55, and 65 kg. We obtained baseline thermodilutions, protocolizing the injection of 30 cc of cold saline into the right cavoatrial junction as described in Box 1. The temperature was sensed in the PA. Simultaneously, flow probes were placed around the main PA, and CO was calculated directly. Using this CO, the correction factor needed to generate that CO from the thermodilution was backed out (Fig 4). Here, the *x*-axis has the CO as directly measured from the flow probes, and the *y*-axis has the correction factor from the thermodilution needed to obtain that CO from the thermodilution. Animals were given norepinephrine, esmolol, and dobutamine to vary their CO across wide ranges to see if the correction factor was sensitive to these physiologic changes. Fig 4 shows the simple linear regression and 95% confidence intervals for this relationship, and the slope was not statistically different from 0 (*P* = .063) across this range, suggesting that in our animal model and thermodilution strategy, a correction factor of 1.0 is reliable across a wide range of COs and physiologic conditions. We further corroborated this model and experimental setup with a sensitivity analysis (Supplementary Methods and Supplementary Fig, online only).

#### Determining load-independent BV PV loop parameters using IVC occlusion

Using the above-described strategy along with the manufacturer guidelines for setting up and using the PV loop systems is sufficient to obtain BV PV loops and load-dependent measures of cardiac function totally endovascularly. Much of the utility, however, of PV loops over other cardiac assessments is the power to obtain preload and afterload independent metrics of cardiac function, namely the end-systolic and end-diastolic pressure-volume relationships and their associated metrics.

**Fig 5:**
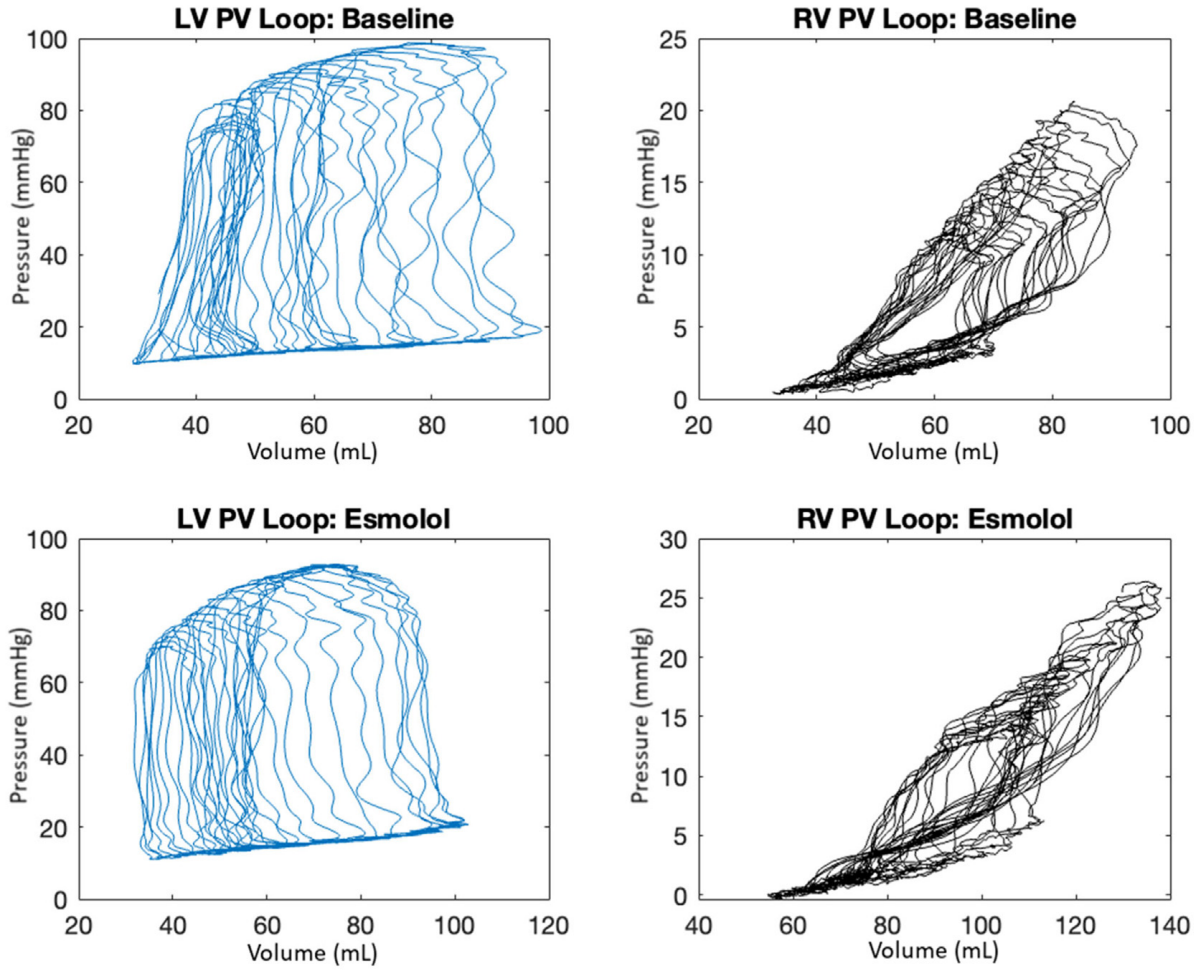
Biventricular (BV) (simultaneously obtained left ventricular [*LV*], blue, and right ventricular [*RV*], black) pressure-volume (*PV*) loops obtained during inferior vena cava (IVC) occlusions, one without physiological modification and one with modification by administration of esmolol. The top two panels show contemporaneous LV and RV, respectively, and PV loops over 5 seconds of IVC occlusion. The bottom two panels show a second IVC occlusion after esmolol has been administered.

These metrics can be obtained by abolishing preload with a temporary IVC occlusion. An example of this, as shown on intraoperative fluoroscopy, is shown in Supplementary Video 2, online only. We have previously published a detailed description of how to analyze LV PV loops over multiple beats with and without IVC occlusion, along with code to derive these metrics,^9^ which are also applicable to PV loops in this scenario. Fig 5 represents an example of this strategy put into practice with two pairs of PV loops. The top two panels are baseline LV (left) and RV (right) contemporaneous PV loops at baseline during an IVC occlusion. Esmolol was administered, and a repeat IVC occlusion was performed.

## Discussion

We have described end-to-end implementation of a completely endovascular, percutaneous technique for obtaining BV PV loops for research, achieving a 100% technical success among our animal models. We describe the surgical and technical challenges and our solutions to obtain endovascular BV PV loops, closed-chest CO, and SV, including validation of the correction factor necessary for thermodilution, and perform IVC occlusion in swine weighing 40 to 70 kg. This article should enable a vascular or endovascular translational research laboratory to implement contemporaneous RV and LV PV loops in swine.

We previously described the technical implementation of LV PV loops and analytic strategies to quantify the LV functional parameters over multiple beats and estimate load-independent parameters without necessarily performing an IVC occlusion.^14^^,^^38^^,^^39^ We extend these techniques to include simultaneous RV and LV PV loops and make the model completely endovascular and percutaneous. Using these techniques, one can perform all critical steps in BV PV loop acquisition (safe LV and RV instrumentation, SV assessment via thermodilution, and endovascular IVC occlusion).

Closed-chest PV loops are advantageous in numerous experimental contexts, such as those where a percutaneously delivered medical device is being tested, where incision size matters, where maintaining chest wall compliance is important, or where simply the operators’ skill set or tools are more amenable to a closed chest. Endovascular-only instrumentation reduces the overall instrumentation of the animal. Unfortunately, the limited complement of Millar catheters designed for large animals are large French (5-7F), do not go over a previously placed wire, and are quite stiff. They do not flex and are not steerable. Coming from the carotid, with little ability to steer, it is difficult to position them in the middle of the ventricle. The high stiffness of these catheters also makes them prone to breakage or damage to the valve or ventricle. New techniques and catheter options are needed to reduce catheter attrition. Until they are developed, these are the most reproducible techniques that limit animal attrition, which we view as an inferior outcome compared with catheter destruction. Lyhne et al^32^ have separately described their technique for closed-chest BV PV loops, including high-quality intraprocedural videos of the steps of their technique. We extend this with additional technical tips, including smaller RV access (5-7F vs 16F), technique and validation of the thermodilution correction factor for large swine, and various technical options for IVC occlusion.

To perform endovascular BV PV loops and obtain load-independent data, one must also be able to perform an IVC occlusion and obtain an accurate SV/CO in an endovascular fashion. Although endovascular IVC occlusion is trivial for a vascular surgeon or endovascular specialist, we describe our two primary methods: the use of (1) a resuscitative endovascular balloon occlusion of the aorta (REBOA) via a 7F femoral venous access^40^ or (2) a Coda balloon via an at least 14F venous access,^41^ positioned across or just above the hepatic veins. We view the REBOA as the favored option in survival surgery due to its small access size, which can easily be removed at the end of the case if the animal is survived.^15^^,^^20^^,^^22^^,^^42^ Swine will not lay flat for hours after the procedure, so the Coda access would probably require a cutdown and closure or ligation for survival experiments. In nonsurvival surgery, we have successfully used both strategies, but we still favor using a REBOA because it is less expensive and has a smaller outer diameter and access size.

Endovascularly obtaining SV and CO is technically easy, and several strategies exist to acquire these values.^43^^,^^44^ An accurate SV is important because it is used to calibrate the volume measurement in the PV system. One reasonable option is to estimate based on animal weight, and this would be useful, especially in questions related to the change in cardiac function, which is reasonable in swine studies where there is no direct translation to humans, making precision less important. Ultrasound examination may be used, but it is expensive, requires techniques not immediately translatable from human to swine anatomy, and has poor interobserver reliability. We use thermodilution with PV access because it can be performed in a validated, standard fashion, and no further instrumentation or additional technology is required.

To test the validity of our experimental setup with regard to correction factor and CO in our animal model of 40 to 70 kg Yorkshire swine, we opened the chest, placed flow probes around the main PA, and backed out the necessary correction factor that provided the correct CO. This showed that using a correction factor of approximately 1.0 in swine across our usual range was appropriate and that this was not dependent on CO. We recommend that other laboratories looking to begin BV or LV PV loops with thermodilution (or for any reason to perform thermodilution) repeat this experiment in their animal model(s) of choice. Thermodilution sensor placement significantly affects the validity of the correction factor. When thermodilution temperature was sensed in the aorta, there was a strong association between CO and the correction factor.

Previous generations of PV-sensing technology made true contemporaneous BV PV loops difficult. However, new technology allows both to be performed simultaneously because they operate at different frequencies and the positioning technology has improved.^14^ In contemporaneous BV PV loops, changes can be observed in both ventricles in situations where the chambers become momentarily decoupled, such as in acute right heart failure due to volume overload, where the RV function changes first, leading to LV dysfunction potentially just minutes later. Changes in interventricular dependence or decoupling also occur with various pharmacologic therapies, procedures, or instrumentation. The capability to conduct simultaneous BV PV loops in research is potentially a watershed moment but needs rigor and a well-described methodology to be fully realized.

Endovascular BV PV loops allow for improved data collection when researching effects on cardiovascular physiology. Because a sternotomy or clamshell thoracotomy is not required, the physiology is not significantly altered, which can ultimately confound peri- and postoperative data. BV PV loops can provide important data regarding the short- and long-term effects of endovascular aortic devices, such as devices used for thoracic or abdominal endovascular aortic repair or resuscitation (eg, REBOA). In addition, this technique has the potential to be translated to humans.^45^ Continuous PV loop data acquisition has been proposed in humans in several clinical scenarios, such as during a transcatheter aortic valve repair or after myocardial infarction for early detection of systolic and/or diastolic dysfunction.^12^

This study has several limitations. First, it is a primer on BV PV loops in swine, focusing on animals weighing 40 to 70 kg. We have used it in swine above this range but have focused on this species and weight range because most of our studies focus on this animal model. If one were to adopt this to other large animals, revalidation of the correction factor needed for thermodilution would be required. This may even be necessary if one uses a different subspecies of swine or those from another region. Secondly, although the gender of the animals was chosen arbitrarily, all included animals were male. This may decrease the generalizability of our findings as it focuses on the anatomy and physiology of male swine; however, the cardiovascular anatomy of swine is the same between males and females, and only some differences are expected regarding PV loops.^46^ This technique has the potential to be used for continuous PV loop measurement in awake animals. Currently, our setup faces challenges related to the sensitivity of pressure catheters and potential issues arising from animal movement. However, future research should study whether this is possible with our current system, possibly by tethering the PV loop setup to the animal, or with a wireless PV loop system. In terms of ease of use, the timing and duration of instrumentation were not formally evaluated; however, the instrumentation of the RV and LV was in the order of minutes. Furthermore, our laboratory comprises general, vascular, cardiothoracic, and endovascular surgeons trained primarily on humans and are adapting human endovascular and surgical skills to this model. Other laboratories, such as those comprising anesthesiologists or interventionalists, may favor adaptions to their skill set, such as swine echocardiogram. The goal of this article, however, is to provide a complete model of endovascular BV PV loops that a trained surgical provider can implement.

## Conclusions

BV PV loops allow for sophisticated measurement of load-dependent and load-independent metrics from both the right and left heart. We describe a comprehensive approach to BV PV loops, including a novel technical approach to a model of endovascular, closed-chest BV PV loops. This may be especially important for studying scenarios where left and right heart functions become decoupled, such as acute right heart failure.

## Author Contributions

Conception and design: DS, MR, RT, JM

Analysis and interpretation: DS, RT, NB, DR, JM

Data collection: DS, MR, CP, AB, JM

Writing the article: DS

Critical revision of the article: DS, MR, CP, AB, RT, NB, DR, JM

Final approval of the article: DS, MR, CP, AB, RT, NB, DR, JM

Statistical analysis: DS, CP

Obtained funding: JM

Overall responsibility: DS, JM

## Disclosures

None.

## Thermodilution sensitivity analysis

To further validate the thermodilution method, we performed a sensitivity analysis by modifying the location in which blood temperature was collected. This approach was identical to the one described above, except that temperature was sensed in the aortic arch instead of the pulmonary artery (PA). We hypothesized that temperature deflection would be delayed, and a smaller correction factor would be needed when sensing in the aortic arch compared with the PA, because the distance between injection and sensing is longer, allowing more time for dilution and transit. Figure 1 shows this delay on LabChart in the top two channels. Here, the PA temperature probe deflects first, followed by deflection of the aortic temperature probe, over a longer period and to a smaller degree.

This analysis revealed a strong negative association (*R*^2^ = 0.78, slope: −0.14 units/L/min; *P* < .0001) (Supplementary Fig, online only). With the temperature probe in the aorta, the correction factor is strongly affected by cardiac output (CO). This finding emphasizes the importance of validating the thermodilution method. A 30-cc cold saline bolus into the right cavoatrial junction provides a robust estimate of CO using a correction factor of 1.0. This thermodilution strategy is reliable for determining CO and stroke volume in an endovascular fashion, which allows the biventricular pressure-volume strategy to remain completely endovascular.

## Appendix

Supplementary Video 1 (online only) Main pulmonary arteriogram, before right ventricular (*RV*) pressure-volume (*PV*) loop catheter placement, with the left ventricular (*LV*) PV-sensing catheter in place.

Supplementary Video 2 (online only) Inferior vena cava (*IVC*) occlusion with the left (*LV*) and right ventricular (*RV*) pressure-volume (*PV*)-sensing catheters in place in a totally endovascular model of PV loop acquisition. Shown is the LV PV-sensing catheter with the tip in the apex, the RV PV-sensing pigtail catheter in the RV, and a Coda balloon in the IVC at the level of the hepatic veins.

Supplementary Fig (online only) A sensitivity analysis that determines the correction factor needed to accurately provide the cardiac output, as measured directly by a main pulmonary artery (*PA*) flow probe, using temperature sensed in the aortic arch and the same 30-cc cold saline bolus injected into the right cavoatrial injection.
