# Supplementary material for: Technical and analytical approach to biventricular pressure-volume loops in swine including a completely endovascular, percutaneous closed-chest large animal model
Source: JVS Vasc Sci. 2024 Jan 17;5:100190. doi: 10.1016/j.jvssci.2024.100190 (PMC10938295; doi:10.1016/j.jvssci.2024.100190)
Supplement: Supplementary Video 1 (online only) — Main pulmonary arteriogram, before right ventricular (RV) pressure-volume (PV) loop catheter placement, with the left ventricular (LV) PV-sensing catheter in place. [file mmc1.docx]

**Supplemental Information:**

*Thermodilution sensitivity analysis*

To further validate the thermodilution method, we performed a sensitivity analysis by modifying the location in which blood temperature was collected. This approach was identical to the above, except that temperature was sensed in the aortic arch instead of the PA. We hypothesized that temperature deflection would be delayed, and a smaller correction factor would be needed when sensing in the aortic arch compared to the PA, because the distance between injection and sensing is longer, allowing more time for dilution and transit. Figure 1 shows this delay on LabChart in the top two channels. Here, the PA temperature probe deflects, then the aortic temperature probe deflects later, over a longer period, and to a smaller degree.

This analysis revealed a strong negative association (R^2^=0.78, slope: -0.14 u/L/min, P<0.0001), Supplemental Figure 1. With the temperature probe in the aorta, the correction factor is strongly affected by CO. This finding emphasizes the importance of validating the thermodilution method. A 30cc cold saline bolus into the right cavoatrial junction provides a robust estimate of CO using a correction factor of 1.0. This thermodilution strategy is reliable for determining CO and SV in an endovascular fashion, which allows the BV PV strategy to remain completely endovascular.


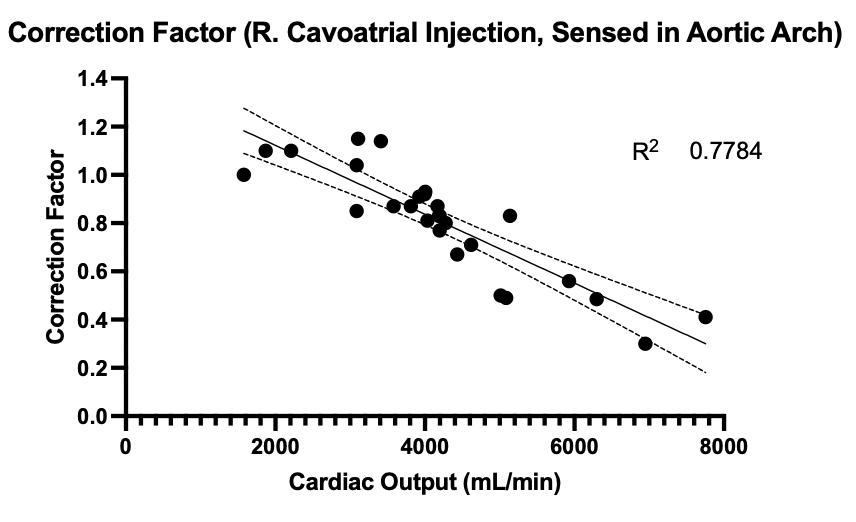


Supplemental Figure 1. A sensitivity analysis that determines the correction factor needed to accurately provide the cardiac output, as measured directly by a main PA flow probe, using temperature sensed in the aortic arch, using the same 30cc. cold saline bolus injected into the right cavoatrial injection.

**Supplemental Video 1.** Main pulmonary arteriogram, prior to RV PV loop catheter placement, with the LV PV sensing catheter in place.

**Supplemental Video 2.** IVC occlusion with the LV and RV PV sensing catheters in place in a totally endovascular model of PV loop acquisition. Shown is the LV PV sensing catheter with the tip in the apex, the RV PV sensing pigtail catheter in the RV, and a Coda balloon in the IVC at the level of the hepatic veins.
